# An experimental hut study to quantify the effect of DDT and airborne pyrethroids on entomological parameters of malaria transmission

**DOI:** 10.1186/1475-2875-13-131

**Published:** 2014-04-01

**Authors:** Sheila B Ogoma, Lena M Lorenz, Hassan Ngonyani, Robert Sangusangu, Mohammed Kitumbukile, Masoudi Kilalangongono, Emmanuel T Simfukwe, Anton Mseka, Edgar Mbeyela, Deogratius Roman, Jason Moore, Katharina Kreppel, Marta F Maia, Sarah J Moore

**Affiliations:** 1 Ifakara Health Institute, Environmental Health and Ecological Sciences, P.O. Box 74, Bagamoyo, Tanzania; 2 London School of Hygiene and Tropical Medicine, Keppel St, London WC1E 7HT, UK; 3 Institute of Biodiversity Animal Health and Comparative Medicine, University of Glasgow, Glasgow G12 8QQ, UK; 4 Swiss Tropical & Public Health Institute, Soccinstraße 57, 4002 Basel, Switzerland; 5 University of Basel, Petersplatz 1, 4003 Basel, Switzerland

## Abstract

**Background:**

Current malaria vector control programmes rely on insecticides with rapid contact toxicity. However, spatial repellents can also be applied to reduce man-vector contact, which might ultimately impact malaria transmission. The aim of this study was to quantify effects of airborne pyrethroids from coils and DDT used an indoor residual spray (IRS) on entomological parameters that influence malaria transmission.

**Methods:**

The effect of Transfluthrin and Metofluthrin coils compared to DDT on house entry, exit and indoor feeding behaviour of *Anopheles gambiae sensu lato* were measured in experimental huts in the field and in the semi-field. Outcomes were deterrence - reduction in house entry of mosquitoes; irritancy or excito-repellency – induced premature exit of mosquitoes; blood feeding inhibition and effect on mosquito fecundity.

**Results:**

Transfluthrin coils, Metofluthrin coils and DDT reduced human vector contact through deterrence by 38%, 30% and 8%, respectively and induced half of the mosquitoes to leave huts before feeding (56%, 55% and 48%, respectively). Almost all mosquitoes inside huts with Metofluthrin and Transfluthrin coils and more than three quarters of mosquitoes in the DDT hut did not feed, almost none laid eggs and 67%, 72% and 70% of all mosquitoes collected from Transfluthrin, Metofluthrin and DDT huts, respectively had died after 24 hours.

**Conclusion:**

This study highlights that airborne pyrethroids and DDT affect a range of anopheline mosquito behaviours that are important parameters in malaria transmission, namely deterrence, irritancy/excito-repellency and blood-feeding inhibition. These effects are in addition to significant toxicity and reduced mosquito fecundity that affect mosquito densities and, therefore, provide community protection against diseases for both users and non-users. Airborne insecticides and freshly applied DDT had similar effects on deterrence, irritancy and feeding inhibition. Therefore, it is suggested that airborne pyrethroids, if delivered in suitable formats, may complement existing mainstream vector control tools.

## Background

Currently, malaria vector control is focused on two interventions: indoor residual spraying (IRS) and long-lasting insecticide-treated nets (LLINs) that have successfully reduced malaria transmission throughout sub-Saharan Africa [1]. In public health vector control programmes, efficacy of insecticidal tools (LLINs and IRS) is measured by the epidemiological endpoints: malaria mortality and morbidity, which can be related to reduced intensity of transmission in the mosquito vector population [2]. Mathematical models have been used to explain dynamics of malaria transmission through entomological, immunological and parasitological parameters that influence malaria transmission [3] expressed as the basic reproductive rate (R_0_). The basic reproductive rate refers to the number of secondary disease infected persons arising from a single infected person in a completely susceptible population [2]. Therefore, the object of any control intervention is to reduce R_0_ to less than 1.

Garrett-Jones [4] described the relationship between entomological parameters that influence malaria transmission, termed the vectorial capacity of a mosquito population. Vectorial capacity equation, subsection). The parameters of the entomological equation include mosquito abundance (m), mosquito daily survival (p) (the vector must live long enough for parasites to develop to the infective stage inside the mosquito) and frequency of contact between mosquitoes and humans through the man biting rate (ma). Vectorial capacity is defined as the expected number of new human malaria infections disseminated per human per day, by a mosquito population from a single case, presuming that all vector females feeding on the case become infective [2].

### Vectorial capacity equation

The vectorial capacity equation as described by Garrett-Jones is as follows: C  = ma^2^p^n^/-log_e_p. C = vectorial capacity, ma = density of mosquitoes per person per night, a^2^ = average frequency of biting on humans (a is squared because a mosquito must bite twice; 1^st^ to receive parasites and 2^nd^ to transmit them), p = the probability of daily survival of the mosquito and n = the duration of sporogony i.e the time required for the parasites to develop in the mosquito (extrinsic period).

According to the vectorial capacity equation, changes to different aspects of the life cycle of mosquitoes will have differential impacts on malaria transmission [5]. For instance, a reduction in mosquito density (m) leads to an equal reduction in vectorial capacity because of their linear relationship, while a reduction in biting rate (ma) leads to a two-fold reduction in transmission due to the quadratic relationship (arising from the fact that mosquitoes need to feed twice to transmit malaria: once to become infected and once to infect) [5]. Importantly, interventions that affect the survival rate (p) of mosquitoes have the greatest impact on transmission due to their exponential relationship [5,6]. Therefore, it becomes obvious why LLINs are such a successful vector control tool: they reduce man-vector contact (ma) because they create a barrier between mosquitoes and humans, reduce mosquito average daily survival (p) through their insecticidal mode of action and therefore also affect mosquito density (m).

Although the primary entomological modes of action (ENMoA) of insecticides used for LLINs and IRS are rapid knockdown and mortality, studies have shown other effects of insecticides that include 1) deterrence: when mosquitoes are prevented from entering human dwellings treated with insecticides [7,8]; 2) irritancy: when mosquitoes contact insecticide surfaces inside houses and leave early [7]; 3) excito-repellency; when mosquitoes contact airborne insecticides and leave the house and 4) feeding inhibition; when mosquitoes are prevented from biting and getting blood meals [7]. The ENMoA of insecticides affect various aspects of the mosquito life cycle and this largely influence the success of any intervention. Despite emphasis placed on the importance of toxic insecticides, studies show that some highly effective insecticides, such as DDT (dichlorodiphenyl trichloroethane), are primarily spatial repellents and feeding inhibitors [9] while toxicity is a lesser, but still important feature [9,10]. In fact, the success of DDT is attributed to its deterrence and irritancy, and only to a lesser extent to its mortality [10,11].

Mosquito coils, vaporizer mats and emanators also induce repellency, irritancy, feeding inhibition and toxicity [12,13]. The impact of coils and emanators on vector borne diseases has been proven. These tools act over a distance by evaporating insecticides into a given space, hence are known as spatial repellents. This mode of action has parallels with the deterrent, feeding inhibition and excito-repellent modes of action of DDT. For this reason, it is worthwhile to compare their effects on entomological components that pertain to vectorial capacity. It is hypothesized that insecticides that have more than one mode of action affect different parameters of the vectorial capacity (m, a, ma, p,) and are likely to bring forth greater changes in transmission than anticipated if only toxicity is considered.

The purpose of this study was to quantify the effect of airborne pyrethroids released by mosquito coils on mosquito behaviour. Emphasis was placed on outcome measures that influence entomological parameters of malaria transmission (Table 1) and to compare the mode of action of transfluthrin and metofluthrin coils against DDT, representing a gold standard insecticide with known impact on malaria transmission [11].

**Table 1:** Entomological parameters of the vectorial capacity targeted by effects of airborne insecticides on mosquito behaviour and the measurement in this study

**Effect of airborne insecticides**	**Parameter of the vectorial capacity**	**System of study**
**Deterrence**	ma^2^	Field
**Excito-repellency and irritancy**	ma^2^	Semi-field
**Toxicity**	m, p	Field and semi-field
**Reduced fecundity (ability of mosquitoes to lay eggs)**	m	Semi-field
**Feeding inhibition (mosquitoes prevented from blood feeding)**	ma^2^	Semi-field

## Methods

Studies were conducted in experimental huts in the field with wild *Anopheles arabiensis* mosquitoes and in a semi-field system [14] with laboratory reared *Anopheles gambiae sensu stricto* (*s.s*.) as a standard test organism for repellents [15]. The overall objective was to determine the effect of DDT, Metofluthrin and Transfluthrin coils on parameters of vectorial capacity using experimental huts.

### Outcomes measured in the field

#### Deterrence

Deterrence refers to reduced house entry of mosquitoes resulting to reduced indoor densities. It was determined by comparing the total number of mosquitoes in huts with insecticides to control huts. The total number of mosquitoes inside huts included: live and dead mosquitoes in exit traps, dead mosquitoes found on the floor as well as mosquitoes found resting inside the hut.

#### Toxicity

Toxicity of coils and DDT was determined by comparing the proportion of dead versus live mosquitoes in insecticide huts to the control huts. Mosquitoes collected from huts were kept for 24 hours in an insectary after which mortality was recorded.

### Outcomes measured in the semi-field

#### Contact irritancy and excito-repellency

Contact irritancy and excito-repellency refer to the rate at which mosquitoes exit huts after physical contact with insecticide treated surfaces or airborne insecticides, respectively. The exit rate is the proportion of female mosquitoes found in the exit traps at the top of every hour compared with the total number found inside huts (resting or dead on the floor) relative to the control hut. The increased or premature exit of mosquitoes is the estimated irritancy or excito-repellency [16] of insecticides used in the house.

#### Toxicity

The number of dead versus live mosquitoes out of those recaptured was compared between huts. Mortality was recorded after 24 hours. The difference in mortality between a control hut (natural mortality) and a treated hut allows assessment of the insecticide-induced mortality [16].

#### Blood feeding inhibition

Feeding inhibition was determined by comparing the number of blood fed versus unfed mosquitoes of total mosquitoes recaptured from huts.

#### Reduced fecundity of mosquitoes

Fecundity was determined by comparing the proportion of blood fed mosquitoes that laid eggs after exposure to different treatments compared to the control. In addition, the total number of eggs laid by each mosquito was determined.

### Experiment 1: field

#### Study area

The study was conducted in Lupiro village in the Kilombero valley in the South East of Tanzania. Annual rainfall ranges between 1200 and 1800 mm with two rainy seasons per year: November to December and January to April. Annual mean temperature ranges between 20-32°C. Communities in Lupiro practice irrigated rice farming that provides suitable mosquito breeding conditions. *Anopheles arabiensis* is the dominant species (>95% of the malaria vector population) with the remainder comprising *Anopheles funestus sensu lato* (*s.l*.) mosquitoes. There is a high density of culicines comprised of *Culex* and *Mansonia* species [17]. A study conducted at the same time and site indicated 100% susceptibility of *An. arabiensis* mosquitoes to World Health Organization recommended doses of DDT and between 95.8% and 90.2% for Permethrin, Lambda cyhalothrin and Deltamethrin [18].

#### Treatments

Mosquito coils were used at a standard dose recommended and approved by the World Health Organization for Pesticides (WHOPES). They included Transfluthrin (0.03%) and Metofluthrin coils (0.00625%). Seventy-five percent pure DDT wettable powder (AVIMA, South Africa) was applied to woven palm leaf mats using Hudson sprayers at 2 g/m^2^ concentration of the active ingredient. DDT was sprayed on mats that could be rotated between huts during experiments. Rotation of treatments between huts is a crucial part of experimental hut study design because it minimizes the spatial bias between huts that often affects relative mosquito density and behaviour.

**Figure 1:**
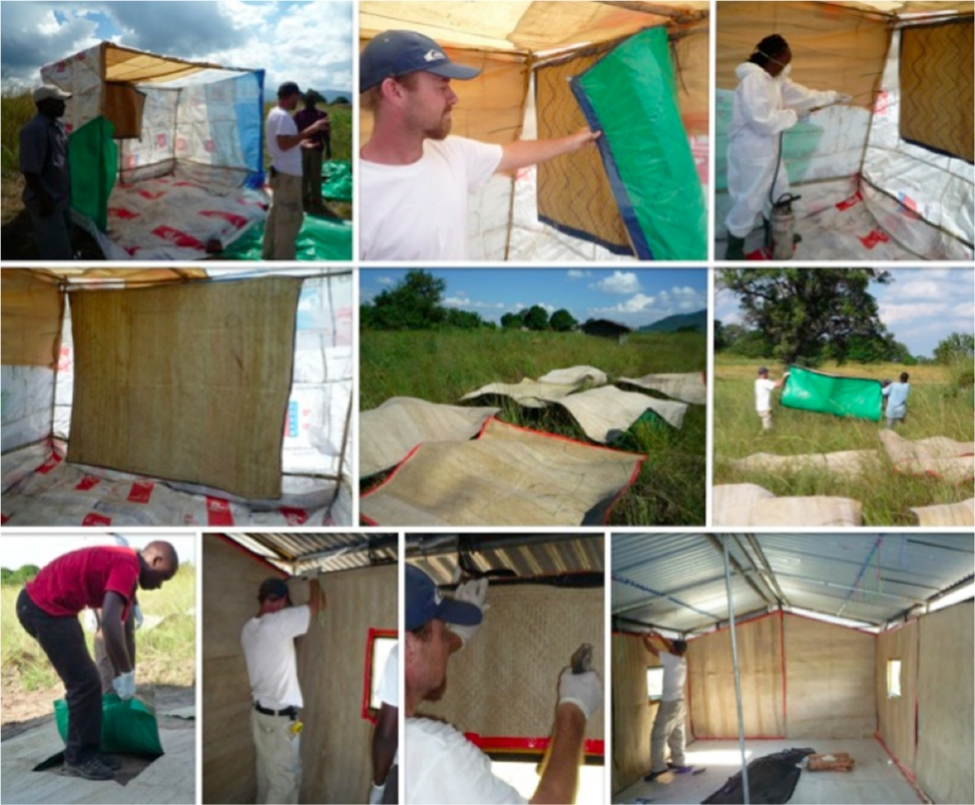
**Spraying palm woven mats with DDT.** Palm woven mats previously cut out to fit on the walls of experimental huts were sprayed with 2 g/m^2^ DDT. Spraying was conducted in a temporary structure that was later burnt. Spraying the mats instead of the walls ensured that mats could be moved easily from one hut to another without contaminating the walls. This allowed rotation of treatments between huts.

Palm woven mats were measured and cut out to fit the entire surface of the inside wall of an experimental hut. The reverse side of the mats was covered with plastic sheets (Figure 1) to prevent contamination of experimental hut surfaces with DDT during rotation of mats between huts. Two sets of mats were prepared, the control was sprayed with water and the other set was sprayed with DDT at a dose of 2 g/m^2^ as recommended by WHOPES [16] using a separate Hudson sprayer for each treatment. The quantity of DDT required to cover walls of one hut was determined by measuring the surface area of walls. The amount of DDT required in g/m^2^ was calculated and weighed. The volume of water required for mixing DDT was determined by pouring a known amount of water in a Hudson sprayer. The sprayers were calibrated to 55 psi and control mats were sprayed with water. The volume of water used in the control was measured and an equal volume of water was used for mixing DDT in a plastic bucket. Spraying was conducted in a disposable tent located 50 metres from experimental huts (Figure 1). The mats were air dried for 15 minutes then fixed to respective walls using removable staples so that they could be detached easily during rotation (Figure 1).

#### Experimental huts

**Figure 2:**
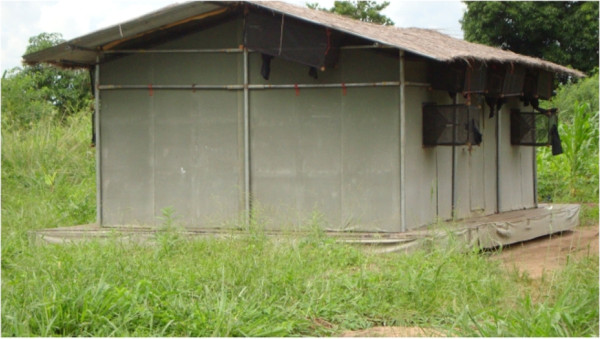
**Ifakara experimental hut.** Experimental huts are representative of local houses found in the study area. The huts are rectangular and similar to most houses within the area. The roof is made of iron sheets and a layer of grass at the top. The walls are made of mud panels and canvas material on the outside.

**Figure 3:**
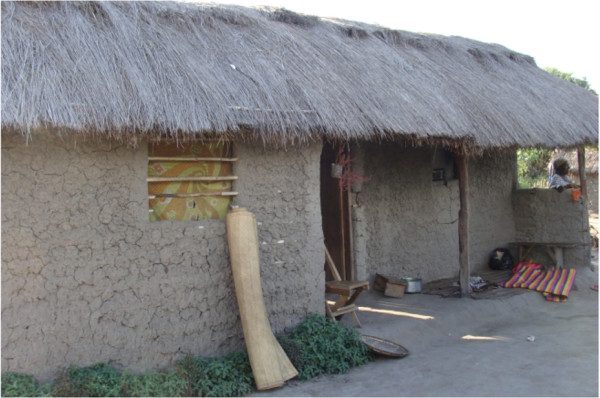
**Local huts.** Local houses in the study area are constructed with corrugated iron sheets or thatched roofing and the walls are constructed with bricks or mud. Most houses are rectangular in shape and have open eaves.

Studies were conducted in Ifakara experimental huts [19] (Figure 2). Initially, information about the size, design of the houses and the materials required for constructing the roofs and walls was collected through a house hold survey conducted in Kilombero valley. The local houses (Figure 3) in this region are constructed with corrugated iron sheets or thatched roofing and walls are constructed with bricks or mud. This information was used in the construction of experimental huts to ensure a good representation of local houses in Kilombero valley. The experimental huts measure 6.5 m long, 3.5 m wide and 2.5 m high at the roof apex. They are made of galvanized pipe framework, the roof is made of corrugated iron sheets and the inner walls are made of removable mud panels while outer walls are covered with canvas. The outer roof is grass thatched. This provides cool temperatures inside huts just like in local houses. Each experimental hut has one door and four windows. The huts have open spaces (eaves) between the roof and the wall similar to local huts. This results in volume, surface area, temperature and air-flow profiles similar to local homes, which is extremely important when measuring spatially active vector control tools. Half of the eaves and all of the windows are fitted with exit traps suspended outside the huts to trap those mosquitoes that attempt to leave. The traps are made of metal frames and UV resistant black plastic coated fibreglass netting (Phifer, USA). The traps are fitted with cotton sleeves through which mosquitoes can be collected. On the eaves there are spaces left between traps. These spaces are fitted with netting baffles through which mosquitoes enter huts but cannot leave. Mosquitoes can only leave through exit traps. Previous studies indicated that entry behaviour of mosquitoes in experimental huts was similar to local houses [17].

#### Study design

A partially-randomized fully-balanced 4 × 4 Latin square design was performed to determine efficacy of DDT used as IRS, Transfluthrin and Metofluthrin coils in four experimental huts. The treatments were tested for four nights per week and were rotated weekly. Therefore, one balanced round of experiments was completed in 16 days. Four rounds of 16 days were performed (n = 64 nights). The treatments tested were: 1) standard control – DDT IRS; 2) negative control – no insecticide used; 3) two Transfluthrin coils (0.03%) per hut each night and 4) two Metofluthrin (0.00625%) coils per hut each night. The huts were located approximately 300 metres from local houses and arranged linearly along a mosquito-breeding site with 50 metres spaces left between them to minimize interaction between treatments. Treatments were randomly allocated to huts with two male volunteers. Treatments were not moved between huts on a nightly basis because of the possibility of a carryover effect of treatments. The huts were left without treatments during the fifth, sixth and seventh night in order to wash out the effect of the previous treatment, after which treatments were moved to the next hut. Two coils were placed on the floor in the middle of the hut at the start of the experiment and they were replaced with new ones when they burnt out. Freshly sprayed DDT mats were used for each round of experiment, meaning that sprayed mats were used for one month and kept in a store to be later burnt in an incinerator.

#### Mosquito collection

Experiments were conducted between 24^th^ November 2010 and 15^th^ October 2011 for 64 nights. Experiments took place each night between 1800 hours and 0600 hours. Every evening, volunteers removed all insects and predators from exit traps to prepare huts for the next experimental night and then they retired to bed. In coil huts, technicians lit two coils and volunteers were given additional coils and instructed to replace those that burnt out before 0600 hours. Volunteers slept under untreated bed nets and woke up at the top of every hour to collect mosquitoes from exit traps. Mosquitoes were collected between 1900 hours and 0600 hours using a mouth aspirator and a spotlight for a maximum of 15 minutes each hour. At 0600 hours, all mosquitoes resting inside the huts as well as those found on the floor were collected. Mosquitoes were placed in paper cups labelled by the time and place of collection (exit traps, resting on hut surfaces and the floor), provided with 10% glucose solution soaked on pieces of cotton wool and kept in a field insectary for 24 hours. Mean temperature inside the insectary was 29.1°C ± 3.0°C during the day and 26.7°C ± 2.3°C at night, while mean relative humidity was 70.6% ± 17.9% during the day and 75.7% ± 13.7% at night. The insectary was located 50 m away from experimental huts.

#### Mosquito handling and identification

Each morning, mosquitoes previously collected from huts and kept for 24 hours in the insectary were morphologically identified as *An. gambiae s.l*., *Mansonia* spp. or *Culex* spp. Mosquitoes were also grouped as either dead, alive, fed or unfed. A sub sample of the *Anopheles* genus mosquitoes was randomly selected and transported to the laboratory for further identification to species using ribosomal DNA-polymerase chain reaction (PCR) [20].

#### Quality control: assessment of the carryover effect of airborne insecticides

During experiments, there was a three-day wash out period after four days of experiments when there were no insecticides in the huts. Volunteers entered huts at 1800 hours and slept until 0600 hours. They collected mosquitoes in exit traps, from resting surfaces inside huts and the floor at 0600 hours. This experiment enabled us to determine whether the three-day wash out period was sufficient to reduce any residual airborne insecticides before treatments were rotated between huts.

#### Assessment of residual efficacy of DDT on grass woven mats

The method of evaluating residual efficacy of DDT on grass woven mats was based on the WHO insecticide testing guidelines [16]. Two locations on each of the four “walls” of DDT sprayed mats were randomly selected. WHO cones were attached on the walls using masking tape and 10 laboratory-reared, 2–6 day old female nulliparous *An. arabiensis* mosquitoes were introduced into each cone. The time was noted and mosquitoes were removed from the cones after 30 minutes. Mosquitoes removed from cones were kept in the field insectary and monitored for 24 hours after which dead and live mosquitoes were recorded. Bioassays were conducted a day after spraying and once every week for four weeks during experiments. Additional control bioassays were conducted simultaneously on control mats previously sprayed with water only.

### Experiment 2: semi-field

#### Semi-field system

**Figure 4:**
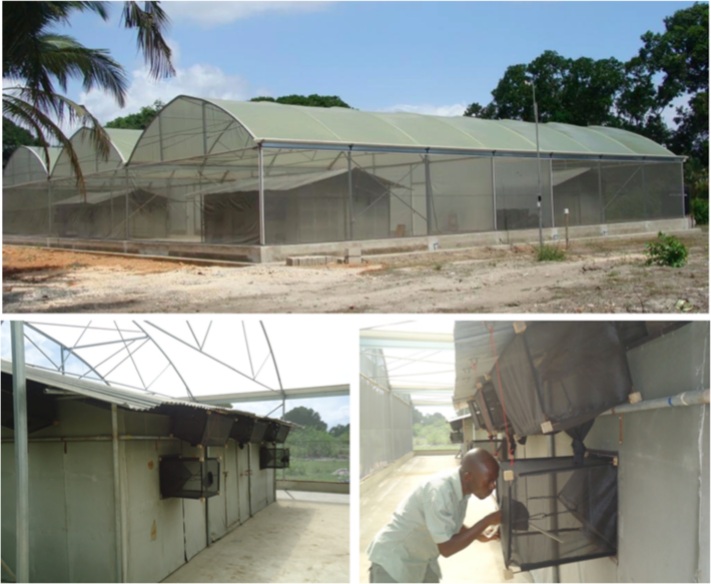
**Semi-field system.** The walls and the roof of the semi-field system (SFS) are made of metal frames and fibreglass netting material. It was divided into four equal square sections divided by fibreglass netting. An experimental hut was placed in each compartment. The SFS [14] allowed replication of experiments within a short period of time. Laboratory reared mosquitoes were used and were available throughout the duration of experiments hence there were no delays as usually experienced in the field.

Studies were conducted in experimental huts placed inside a Semi-Field System (SFS) in Bagamoyo District, Tanzania (Figure 4). Use of the SFS [14] allowed replications of experiments within a short period of time because laboratory reared mosquitoes were used and therefore experiments were not dependent on the season. In addition, laboratory mosquitoes are disease free, therefore, not putting volunteers at risk of being infected with mosquito-borne diseases.

#### Mosquitoes

Insecticide susceptible mosquitoes of the species *An. gambiae s.s*. (Ifakara strain) were used. The colony was maintained by feeding larvae on Tetramin fish food and adults on human blood between 3 and 6 days after emergence and 10% glucose solution *ad libitum*. Temperature and humidity within the insectary were maintained between 28 – 29°C and 70 - 80% respectively. The mosquitoes used in the experiments were female nulliparous, 3–8 days old *An. gambiae s.s*. that had never blood fed and were sugar starved for 6 hours prior to the start of experiments.

#### Study design

**Figure 5:**
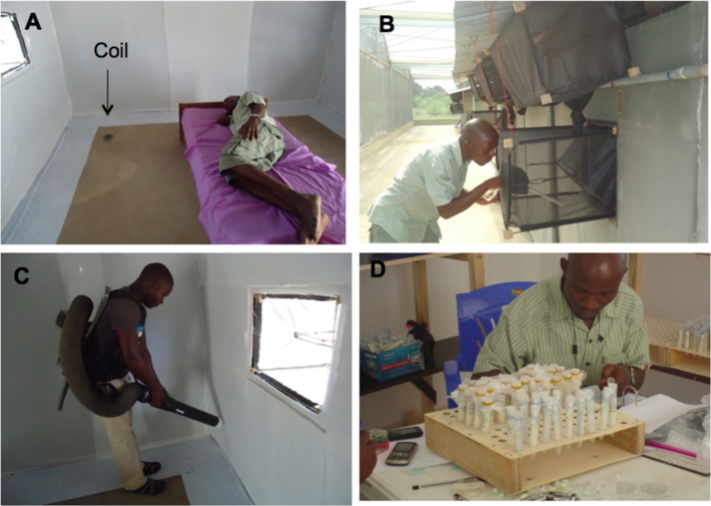
**Process of collecting mosquitoes from experimental huts. A:** A coil placed on the floor 0.5 m from the volunteer **B:** HN collecting mosquitoes from exit traps using a mouth aspirator; **C:** AM collecting resting mosquitoes using a backpack aspirator; **D:** HN sorting mosquitoes and keeping them in individual tubes for checking oviposition.

Four Ifakara design experimental huts (Figure 2) fitted with window and eave exit traps were used inside the SFS. The huts were placed in individual compartments separated by 10 metres and a netting screen. A fully-randomized fully-balanced 4 × 4 Latin square design was performed to determine efficacy of DDT used as IRS, Transfluthrin and Metofluthrin coils in four experimental huts. The treatments were tested for four nights per week. Therefore, one balanced round of experiments was completed in 16 days. The treatments tested were: 1) standard control – DDT as IRS; 2) negative control – no insecticide used; 3) two Transfluthrin coils (0.03%) per hut each night and 4) two Metofluthrin (0.00625%) coils per hut each night. Treatments and two male volunteers were randomly allocated to each hut. The pair of volunteers was rotated between huts every fourth night while the treatments remained in the same huts during the entire study period. Equal numbers of mosquitoes were used in each compartment, hence there was no need to rotate the treatments between huts to minimize location bias as is the case in field experiment. Experiments began each evening at 1930 hours when volunteers entered respective huts. Technicians placed two lit coils on the floor 0.5 m from the volunteer inside respective huts (Figure 5A). After 10 minutes, the volunteers simultaneously released 100 female mosquitoes in each hut from netting cages. The volunteers slept on mattresses on the floor and did not use bed nets.

#### Mosquito collection and processing

Technicians collected mosquitoes from exit traps at the top of every hour from 2100 hours to 0700 hours using mouth aspirators (Figure 5B, 5). Additional collection was done at 0700 hours inside the huts to capture resting, knocked down and dead mosquitoes using CDC backpack aspirators (Figure 5C). Mosquitoes were placed in labelled paper cups and provided with 10% glucose solution. They were kept in an insectary with temperature at 28 – 29°C and between 70 - 80% relative humidity. Each morning mosquitoes were sorted as either dead or alive, and fed and unfed. The total number of mosquitoes in each group was recorded. Blood fed mosquitoes were kept in the insectary in individual vials with moist filter paper and were left to lay eggs (Figure 5D). The number of eggs in each vial was counted and recorded after 3 days.

#### Protection of participants and ethical approval

The male persons who slept in experimental huts were recruited on a voluntary basis through written informed consent after the risks and benefits of the study were clearly explained, and they were free to leave at any time during the study. The participants were screened for malaria before the beginning of the study and those participants found malaria positive were given artemisinin combination therapy anti-malarial drugs and referred to the nearest health centre. Those fit to participate in the study were tested for malaria every two weeks. Adverse events such as respiratory symptoms were monitored. The participants were also compensated for their time and effort. The ethical review boards of Ifakara Health Institute IHI/IRB/No A-019-2007, the National Malaria Research Institute Tanzania (NIMR/HQ/R.8a/Vol.1X/710) and the London School of Hygiene and Tropical Medicine (LSHTM ERB 5552) approved the study.

#### Statistical analysis

Some of the data was analysed using the R statistical software version 3.02 [21] with significance level of 0.05 for rejecting the null hypothesis. All generalized linear mixed models (GLMMs) were conducted using the lme4 package [22].

#### Assessment of residual efficacy of DDT on grass woven mats

Mortality of mosquitoes in different cone assays was calculated as a proportion of the total number of those exposed to the chemical.

#### Deterrence

Deterrence was determined using GLMMs. The model included the number of mosquitoes as the response variable (dependent variable) and the independent variables included the hut and treatment as fixed factors and the day of experiment as a random variable. The first model did not account for overdispersion in the data (performing a Poisson GLMM), the second model accounted for overdispersion by fitting a random intercept for each row of the data (performing a log-normal Poisson GLMM) and the third model was fitted with an interaction term between hut and treatment and accounted for overdispersion. The models were compared using Aikaike’s Information Criterion (AIC) [23] and the second model was chosen because it had the smallest AIC.

#### Toxicity

The proportion of mortality in the field study was calculated using the following formula: 100 × (Dt–Dc)/Ec (The proportion of dead mosquitoes Dt = number of mosquitoes dead in treated hut, Dc = number of mosquitoes dead in control hut and Ec = total number of mosquitoes in control hut [15]). Mortality in the semi-field studies was determined by fitting a GLMM with binomial error and a logit link function. The dependent variable was the proportion of dead mosquitoes and independent variables were treatment and trap (exit or floor or resting) included as fixed factors while the day of experiment was set as a random variable.

#### Contact irritancy and excito-repellency

The number of mosquitoes that exited huts was compared to those that stayed inside the huts that had insecticides relative to the control. A GLMM with a binomial error and a logit link function was fitted. The dependent variable was the proportion of exiting mosquitoes. Independent variables included treatment as fixed factor and day as a random factor.

The rate at which mosquitoes left huts that had insecticides was compared to the control huts using survival analysis and Kaplan-Meier survival graphs. Analysis was conducted with survival and splines survival packages in R. The time at which an individual mosquito left the hut was considered to be the “event”.

#### Blood feeding inhibition

The proportion of blood-fed mosquitoes was compared between the treatment and control huts in the semi-field experiments. This was determined by fitting a GLMM with binomial error and logit link. The dependent variable was the proportion of unfed mosquitoes and independent variables included treatment, volunteer and trap type as fixed factors and day as a random variable.

#### Reduced fecundity

The data was analysed in two different ways. The first method was to determine the proportion of mosquitoes that laid eggs after blood feeding in the presence of insecticides in semi-field experiments. This was determined by fitting a GLMM with binomial error and logit link. Treatment was included as a fixed factor and day of experiment as a random variable.

The second method was used to determine the number of eggs laid by blood fed mosquitoes exposed to insecticides compared to the control. The effect on number of eggs laid was determined using a GLMM. A Poisson model was fitted with the number of eggs as the dependent variable and the independent variables included treatment as a fixed factor and the day of experiment as a random variable. The best fitting model as measured by AIC did not account for overdispersion.

## Results

### Experiment 1 field

The total number of mosquitoes collected was 30,280 of which 19,593 mosquitoes were *An. gambiae s.l*., 2016 were *Mansonia* sp. 7829 were *Culex quinquefasciatus*, 136 were *Stegomyia* aegypti [24] and 706 were *Anopheles coustani*. PCR analysis was conducted on species of *An. gambiae s.l*., 100% (n = 975) of all successful amplifications were *An. arabiensis* mosquitoes.

### Quality control: assessment of the carryover effect of airborne insecticides

During the three-day wash period, the total number of mosquitoes inside huts increased gradually from the first day to the third but there was no significant difference between the days (Table 2). There was no significant difference in the number of mosquitoes between huts that previously contained insecticides and the control hut (Table 3).

**Table 2:** Total mosquitoes collected from experimental huts in the field during the 3 – day wash out period (experimental nights; n = 12)

**Day of wash out**	**N**	**Median**	**IQR**	**RR**	**95% CI**	**z value**	**p value**
**1**	1064	42.0	23.8 – 96.3	NA	NA	11.168	NA
**2**	1238	46.0	34.5 – 100.8	50.3	[20.2 - 125.5]	0.240	0.810
**3**	1187	59.0	52.0 – 91.5	54.8	[22.0 - 136.7]	0.425	0.671

Legend: This table illustrates the indoor densities of mosquitoes of experimental hust that previously had coils and DDT. Entry of mosquitoes was measured for 3 days. N - Total number of mosquitoes; Median – Number of mosquitoes per experimental day; IQR – Interquartile range; RR – Relative rate CI – Confidence intervals.

**Table 3:** Total mosquitoes that entered untreated huts that previously had insecticides (experimental nights; n = 12)

**Treatment**	**N**	**Median**	**IQR**	**RR**	**95% CI**	**z value**	**p value**
No insecticide	1054	47.0	22.0 – 110.3	45.0	[23.1, 87.8]	11.168	NA
Transfluthrin coils	737	71.0	28.0 – 92.5	41.1	[33.6, 50.2]	-0.897	0.369
Metofluthrin coils	877	67.0	39.8 – 103.0	51.1	[42.0, 62.3]	1.273	0.203
DDT 2gm^2^	821	42.5	37.5 – 72.3	44.9	[36.8, 54.8]	-0.014	0.989

Legend: This table illustrates indoor mosquito densities in huts that previously had coils and DDT. The mosquitoes were collected during the wash period when the huts had no insecticides.

N = Total number of mosquitoes; Median = Number of mosquitoes per hut per night; IQR – Interquartile range; RR – Relative rate CI = Confidence intervals.

### Deterrence

All compounds deterred malaria vectors from entering huts but coils had a greater impact than DDT (Table 4). Transfluthrin coils reduced entry of *An. arabiensis* mosquitoes by 38% (RR – 0.62 [0.47 - 0.87]; z = -6.37, p < 0.001). Metofluthrin coils reduced *An. arabiensis* mosquitoes by 30% (RR – 0.70 [0.50 - 0.98]; z = -4.77, p < 0.001) while DDT reduced them by 8% (RR – 0.92 [0.65 - 1.20]; z = -1.22, p = 0.224) (Table 4). Both Metofluthrin and Transfluthrin coils reduced entry of *Mansonia* spp. mosquitoes by more than three quarters while DDT reduced them by half (Table 3). There was no significant difference in the number of *Cx. quinquefasciatus* mosquitoes entering control, DDT and Transfluthrin huts although Metofluthrin coils did reduce their entry.

**Table 4:** Indoor mosquito densities in field experimental huts that had mosquito coils and DDT compared to huts that did not have insecticides (n = 64 nights)

**Treatment**	**N**	**Median**	**IQR**	**RR**	**95% CI**	**z value**	**p value**
***Anopheles arabiensis***							
No insecticide	5650	70.00	50.25 – 104.50	NA	NA	NA	NA
Transfluthrin coils	3881	47.00	27.25 – 75.25	0.62	[0.47 - 0.87]	-6.37	<0.001
Metofluthrin coils	4249	54.00	35.50 – 82.00	0.70	[0.50 - 0.98]	-4.77	<0.001
DDT 2gm^2^	5813	67.00	41.50 – 108.75	0.92	[0.65 - 1.20]	-1.22	0.224
***Culex quinquefasciatus***							
No insecticide	2300	26.00	19.50 – 46.25	NA	NA	NA	NA
Transfluthrin coils	1782	26.50	13.00 – 39.25	0.87	[0.73 - 1.05]	-1.46	0.143
Metofluthrin coils	1645	22.50	13.75 – 36.25	0.72	[0.61 - 0.85]	-3.80	<0.001
DDT 2gm^2^	2102	27.00	16.75 – 44.00	1.13	[1.01 - 1.28]	-1.40	0.161
***Mansonia*** **spp.**							
No insecticide	947	12.00	8.75	NA	NA	NA	NA
Transfluthrin coils	150	2.00	1.00	0.16	[0.07 - 0.19]	-8.17	<0.001
Metofluthrin coils	185	2.00	0.75	0.12	[0.09 - 0.24]	-7.56	<0.001
DDT 2gm^2^	734	9.00	5.75	0.50	[0.33 - 0.77]	-3.16	0.002

Legend: The table illustrates reduction of indoor mosquitoes when huts were treated with coils and DDT, N- Total number of mosquitoes; Median – number of mosquitoes per hut per night; IQR – Interquartile range; RR – Relative rate; CI – Confidence intervals.

### Toxicity

Mortality of mosquitoes after 24 hours in field experiments was very low. Only 0.02% mortality of all mosquito species collected was observed.

### Residual efficacy of DDT on grass woven mats

Cone bioassays conducted on DDT mats on the second day and a week after spraying showed 100% mortality of mosquitoes after 24 hours. Mortality dropped in the second, third and fourth week to 73%, 92% and 90%, respectively. It is likely that DDT flaked off from mats when they were moved between huts resulting in reduced residues hence reduced toxicity. There was no mortality in the bioassays conducted on control mats.

### Experiment 2: semi-field

Seventy percent (n = 4476/6400) of the mosquitoes released in the huts were recaptured. The relatively low recovery rate could be explained by loss of mosquitoes that might have been eaten by predators and those that escaped through small cracks in the huts or when the door was opened briefly. However analysis was conducted on recovered mosquitoes and not released mosquitoes.

### Contact irritancy and excito-repellency

**Figure 6:**
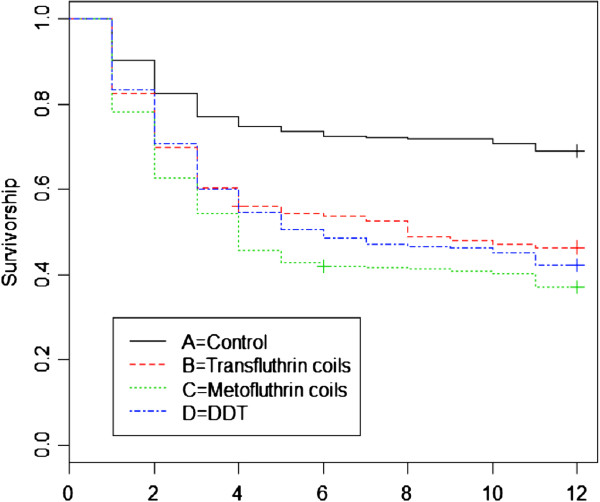
**Survival curves illustrating the rate at which mosquitoes left huts with DDT, transfluthrin and metofluthrin coils.** The curves represent the rate at which mosquitoes exit huts that have different insecticides compared to the control. Time (hours) to which mosquitoes left huts: 1–2100, 2–2200, 3–2300, 4–0000, 5–0100, 6–0200, 7–0300, 8–0400, 9–0500, 10–0600, 11–0700. Analysis was based on a Kaplan-Meier stepped survivorship function. Each curve represents one treatment.

The proportion of mosquitoes that left huts that had DDT, Transfluthrin and Metofluthrin coils was significantly higher than the control (Table 5). Approximately 48% (95% CI: [0.44 -0.53]; z = 9.950, p < 0.001) of the mosquitoes left DDT huts (Table 5). In huts with Transfluthrin and Metofluthrin coils approximately 56% (95% CI: [0.51 - 0.60]; z = 12.779, p < 0.001) and 55% (95% CI: [0.51 -0.60]; z = 12.890, p < 0.001) left huts, respectively. The rate at which mosquitoes left huts throughout the night is illustrated using Kaplan Meier survival curves (Figure 6). The highest exodus of mosquitoes from huts was observed in the first half of the night (2100 – 0000 hours) regardless of treatment or control, but overall, more mosquitoes exited when huts contained DDT, Transfluthrin or Metofluthrin coils compared to the control.

**Table 5:** The proportion of the mortality of mosquitoes 24 hours after collection from experimental huts

**Treatment**	**Total dead mosquitoes**	**Total mosquitoes recaptured**	**Crude mortality**		**OR [95% CI]**	**Corrected mortality**^⌘^		**z value**	**p value**
			**Mean proportion**	**[95% CI]**		**Mean proportion**	**[95% CI]**		
**Control**	193	1067	0.17	[0.13 - 0.22]	1.00 [0.00 – 2.00]	0.00	[0.00 - 0.00]	-10.04	NA
**DDT**	836	1185	0.70	[0.65 - 0.74]	9.68 [4.19 – 21.00]	0.64	[0.60 - 0.67]	22.49	<0.001
**Metofluthrin coils**	763	1157	0.67	[0.63 - 0.72]	8.77 [2.34 – 17.79]	0.61	[0.57 - 0.64]	21.96	<0.001
**Transfluthrin coils**	727	1067	0.72	[0.68 - 0.76]	10.85 [1.53 – 21.01]	0.66	[0.63 - 0.70]	23.32	<0.001

Legend: Experiments were conducted in experimental huts within a semi field system. Mortality of mosquitoes is compared between huts that had mosquito coils, DDT and no insecticide. Experiments were conducted for 16 nights. CI – Confidence intervals. ⌘ - Corrected using Abbott’s formula; OR – Odds ratios of the proportion of mosquitoes.

### Toxicity

The proportion of mortality in control huts was 18% (n = 193/1067). Therefore, Abbot’s correction formula was used to correct for mortality induced by tested insecticides because mortality in the control huts was more than 10% [15]. There was a much higher proportion of mortality induced by insecticides in the semi-field study compared to the field. DDT induced 64% (95% CI: [0.60 - 0.67]; z = 22.49, p < 0.001), Transfluthrin induced 66% (95% CI: [0.63 - 0.70] z = 23.32, p < 0.001) and Metofluthrin 61% (95% CI: [0.57 - 0.64]; p < 0.001; z = 21.96) mortality (Table 6). More than 90% of the mosquitoes collected inside huts that had mosquito coils and DDT had died within 24 hours unlike in the control hut (Table 7). Out of the mosquitoes collected from exit traps of DDT Transfluthrin and Metofluthrin huts, 49%, 46% and 57%, respectively died after 24 hours (Table 7).

**Table 6:** Mortality of mosquitoes collected from exit traps compared to those collected inside experimental huts

**Treatment**	**Mosquitoes in exit traps**			**Mosquitoes indoors**		
	**Dead mosquitoes/Total mosquitoes**	**Median**	**IQR**	**Dead mosquitoes/Total mosquitoes**	**Median**^**2**^	**IQR**
**Control**	91/313	0.35	0.19 – 0.43	102/754	0.15	0.09 – 0.18
**DDT**	286/581	0.52	0.30 – 0.75	550/604	1.00	0.94 – 1.00
**Transfluthrin coils**	273/599	0.49	0.34 – 0.55	454/468	1.00	1.00 – 1.00
**Metofluthrin coils**	333/645	0.35	0.25 – 0.73	430/512	1.00	1.00 – 1.00

Legend: The proportion of mortality induced by insecticides was measured in experimental huts in a semi field system for 16 nights. Mortality was compared between huts that had mosquito coils, DDT and no insecticides.

Median – median proportion of mosquitoes per hut per night; IQR – Interquartile range.

**Table 7:** Mortality of mosquitoes collected from exit traps compared to those collected inside experimental huts

**Treatment**	**Mosquitoes in exit traps**			**Mosquitoes indoors**		
	**Dead mosquitoes/total mosquitoes**	**Median**^**1**^	**IQR**	**Dead mosquitoes/total mosquitoes**	**Median**^**2**^	**IQR**
**Control**	91/313	0.35	0.19 – 0.43	102/754	0.15	0.09 – 0.18
**DDT**	286/581	0.52	0.30 – 0.75	550/604	1.00	0.94 – 1.00
**Transfluthrin coils**	273/599	0.49	0.34 – 0.55	454/468	1.00	1.00 – 1.00
**Metofluthrin coils**	333/581	0.35	0.25 – 0.73	430/512	1.00	1.00 – 1.00

Legend: The proportion of mortality induced by insecticides was measured in experimental huts in a semi field system for 16 nights. Mortality was compared between huts that had mosquito coils, DDT and no insecticides.

Median^1^ – median proportion of mosquitoes in exit traps per hut per night; IQR – Interquartile range; Median^2^ – median proportion of mosquitoes inside each hut per night.

### Blood feeding inhibition

Blood-feeding inhibition was the most pronounced mode of action in all three treatments. Transfluthrin and Metofluthrin coils had the highest impact on feeding of mosquitoes. Transfluthrin coils reduced feeding by 98% (95% CI: [0.96 - 0.99]; z = 22.03, p < 0.001), Metofluthrin reduced it by 93% (95% CI: [0.90 – 0.95]; z = 25.57, p < 0.001) and DDT by 77% (95% CI: [0.73 - 0.81]; z = 24.10, p < 0.001) (Table 8).

**Table 8:** Insecticide induced blood-feeding inhibition of mosquitoes in experimental huts

**Treatment**	**Proportion of unfed mosquitoes**^**a**^	**OR [95% CI]**	**Mean proportion**	**[95% CI]**	**z value**	**p value**
**Control**	321/1120	1.00 [0.00 – 2.00]	0.15	[0.10 - 0.22]	-7.49	NA
**DDT**	881/1047	13.21 [9.96 – 29.04]	0.77	[0.73 - 0.81]	24.10	<0.001
**Transfluthrin coils**	1164/1184	144.87 [67.19 – 382.05]	0.98	[0.96 - 0.99]	22.03	<0.001
**Metofluthrin coils**	1085/1146	44.27 [37.03 – 110.97]	0.93	[0.90 – 0.95]	25.57	<0.001

Legend: The proportion of mosquitos that were inhibited from blood feeding on humans was measured inside experimental huts in a semi field system for 16 nights. The proportion of unfed mosquitoes was compared between mosquito coils, DDT and no insecticide.

^a^unfed mosquitoes/total number of mosquitoes recaptured from the hut; OR – Odds ratios of the proportion of mosquitoes that are likely not to feed; CI – Confidence intervals.

### Reduced fecundity

The difference in the number of mosquitoes that laid eggs versus those that did not lay eggs was determined from the number that acquired blood meals. The proportion of mosquitoes that laid eggs was low in all huts (Table 9). There was no difference in the proportion of mosquitoes that laid eggs between treatments relative to the control. DDT reduced the total number of eggs laid per female by 90% (RR – 0.10 [0.04 - 0.26]; z = -4.57, p < 0.001), Transfluthrin coils by 97% (RR – 0.03 [0.01 - 0.15]; z = -4.13, p < 0.001 and Metofluthrin coils by 91% (RR – 0.09 [0.03 - 0.27]; p < 0.001; z = -4.28) (Table 10).

**Table 9:** The fecundity of mosquitoes after exposure to mosquito coils and DDT in experimental huts

**Treatment**	**Total mosquitoes that laid eggs/total blood fed mosquitoes**	**OR [95% CI]**	**Mean proportion**	**[95% CI]**	**z value**	**p value**
**Control**	202/614	1.00 [0.00 – 2.00]	0.33	[0.28 - 0.37]	-7.36	NA
**DDT**	19/76	0.68 [-0.32 – 1.41]	0.20	[0.13 - 0.30]	-2.41	0.016
**Transfluthrin coils**	1/6	0.41 [-0.36 – 3.02]	0.15	[0.02 - 0.61]	-0.94	0.347
**Metofluthrin coils**	11/34	0.96 [-0.57 – 5.98]	0.24	[0.13 - 0.40]	-1.13	0.258

Legend: Fecundity was measured by determining the proportion of mosquitoes that laid eggs out of those that successfully blood fed. Fecundity was compared between mosquitoes exposed to mosquito coils, DDT and no insecticide inside experimental huts in the semi field system for 16 nights.

CI – Confidence intervals; OR – Odds ratios of the proportion of mosquitoes.

**Table 10:** The proportion of eggs laid by mosquitoes collected from experimental huts

**Treatment**	**Total number of eggs**	**Median**	**IQR**	**RR**	**[95% CI]**	**z value**	**p value**
**Control**	10089	649.0	443.5 - 943.0	NA	NA	18.66	NA
**Transfluthrin coils**	57	0.0	0.0 - 0.0	0.03	[0.01 – 0.15]	-4.13	<0.001
**Metofluthrin coils**	526	0.0	0.0 - 57.5	0.09	[0.03 - 0.27]	-4.28	<0.001
**DDT 2 gm**^**2**^	837	42.0	6.0 - 82.5	0.10	[0.04 - 0.26]	-4.57	<0.001

Blood fed mosquitoes collected from huts that had mosquito coils, DDT and no insecticides were kept in individual oviposition tubes and the number of eggs laid was compare between mosquitoes that had been collected from huts that had different insecticides.

Legend: Median – Total mosquitoes caught per hut; IQR – Interquartile range; RR – Relative rate; CI – Confidence intervals.

## Discussion

Traditionally, efficacy of insecticides for disease control is attributed to toxicity while other effects are considered less important. The spread of insecticide resistance threatens the sustainability of insecticides applied to kill mosquitoes [25,26]. While development of new insecticides is an undisputed requirement to fight insecticide resistance, management of existing insecticides to prolong their usefulness is also necessary.

**Figure 7:**
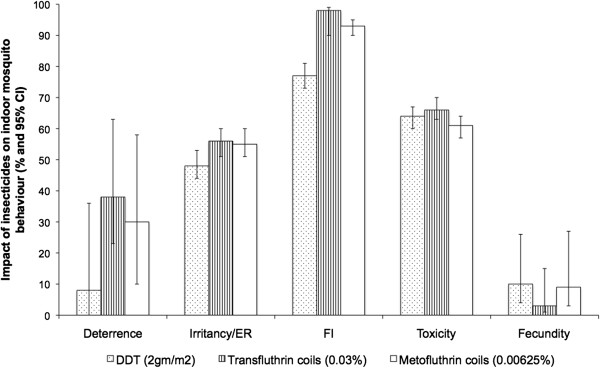
**Overall impact of insecticides on mosquito behaviour insides houses.** The graph illustrates the mode of action of DDT, Transfluthrin and Metofluthrin coils on mosquito behaviour. The outcomes measured included deterrence, irritancy, feeding inhibition and toxicity. The value of deterrence was derived from the effect of insecticides on *An. arabiensis* mosquitoes from field experiments and irritancy, feeding inhibition, mortality and fecundity of *An. gambiae s.s*. mosquitoes from the semi field system experiment.

A critical look at the modes of action of insecticides by several authors indicate that toxicity may not be the single most important action of insecticides as far as malaria transmission is concerned [7,10,27,28]. Experimental hut studies enable detailed observation of the impact of insecticides on mosquito behaviour [29,30]. This study substantiates the mode of action of reduced blood feeding by mosquitoes [9] and irritancy [7,31] (Figure 7). It is worth noting that despite the irritant effect of chemicals, 49% 46% and 57% of the mosquitoes that left DDT, Transfluthrin and Metofluthrin huts respectively died after 24 hours (Table 7). Moreover this study shows that the magnitude of these effects was similar between coils and DDT (Figure 7).

**Figure 8:**
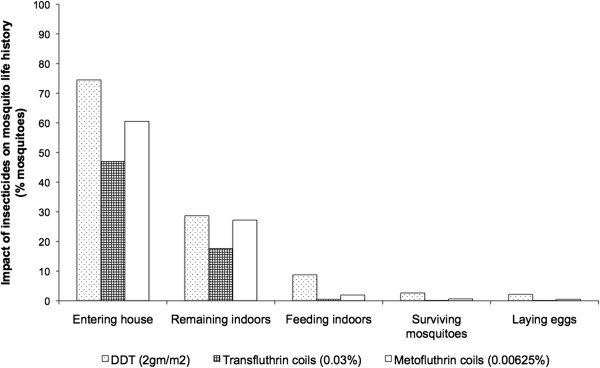
**Impact of insecticides on mosquito behaviour around and insides houses.** The graph illustrates the effect of DDT, Transfluthrin and Metofluthrin coils on the house entry and behaviour of 100 female *An. arabiensis* and *An. gambiae s.s*. mosquitoes are approaching the house. Assumptions made included the fact that deterrence was the first mode of action followed by irritancy, feeding inhibition, toxicity and fecundity. The data used was derived from field experiments for deterrence and semi-field system experiments for irritancy, feeding inhibition, toxicity and fecundity.

Using figures collected from the field (deterrence) and the semi field experiments (irritancy, feeding inhibition, mortality and fecundity) it can be seen that in a scenario where 100 mosquitoes approach a house, deterrence comes into play in the first instance and only approximately 62, 70 and 92 mosquitoes enter the house with Transfluthrin, Metofluthrin coils and DDT respectively. The next behavioural effect of the insecticides is then likely to be irritancy or excito–repellency. After mosquitoes are repelled and exit a house, 35, 39 and 44 would remain inside the house with Transfluthrin, Metofluthrin coils and DDT respectively. Of those, approximately 1, 3 and 10 mosquitoes would manage to acquire a blood meal, which in turn directly influences the proportion of eggs laid, i.e. female mosquito fecundity. Lastly, the survival rate of mosquitoes in Transfluthrin and Metofluthrin huts would be close to 0 and approximately 10 in DDT huts (Figure 8). This implies that through deterrence, irritancy and feeding inhibition of pyrethroid coils and DDT, more than 90% of the mosquitoes would be prevented from contacting humans inside houses before mortality is even considered. By reducing human-vector contact, coils and DDT directly influence the biting rate of mosquitoes (ma): an important parameter of malaria transmission Vectorial capacity equation, subsection). The data collected on DDT, agrees with field observations [9] of feeding inhibition and population level data that consistently demonstrate a reduction in the Human Blood Index (HBI) after DDT is applied to dwellings [32,33]. However, the experimental design does have the limitation of combining data from two species: *An. arabiensis* and *An. gambiae s.s*.

Studies have been conducted on the host preference and time and place of biting and resting in Kilombero. It is known that *An. arabiensis*, the dominant *Anopheles* species in Kilombero, readily enter houses [17], and exit to rest outside whereas *An. gambiae s.s*. feed and rest indoors (K. Kreppel, unpublished). The human blood index (HBI) of *An. arabiensis* is related to the availability of human hosts, and since cattle are not common in Kilombero due to the Tanzanian Government forcibly relocating Pastoralists and their 250,000 cattle, *An. arabiensis* feeds almost exclusively on humans in the area, indoors and outdoors (K. Kreppel, unpublished). As the impact of spatial repellents indoors was being measured, a standard laboratory strain of *An. gambiae s.s*. for repellent testing was used [15]. Previous unpublished work in local houses demonstrated that *An. arabiensis* demonstrated a similar response to 0.03% Transfluthrin coils as that measured in experimental huts with >95% feeding inhibition as measured by human landing catch. It is possible that mortality data was overestimated because *An. arabiensis* might be more likely to leave treated huts than *An. gambiae s*,*s*., although the vast majority of *An. gambiae s.s*. in the semi-field did leave experimental huts unfed and subsequently died. It would be worthwhile to repeat the study with *An. arabiensis*, mosquitoes.

Coils and DDT induced more than two-thirds mortality of mosquitoes in the semi-field experiments compared to about 2% in the field. The mortality (18%) observed in control huts may be attributed to poor handling of mosquitoes during collection. Resting mosquitoes were collected using backpack aspirators that may have caused mechanical damage to mosquitoes and increased mortality. However, mortality in the treatments was corrected using Abbots formula. Higher mortality observed in semi-field experiments compared to the field experiments may be due to the fact that in the semi-field studies, volunteers did not sleep under bed nets and were consequently more attractive to host seeking mosquitoes that spent more time around the host trying to feed. In the field where volunteers were protected by untreated bed nets mosquitoes may have given up and left the huts. It is possible that availability of an unprotected host and the need to obtain blood outweighs the irritant or excito-repellency effects of insecticides, meaning that mosquitoes spend more time in the house trying to obtain a blood meal, hence acquire more lethal insecticides. These observations provide useful insights for malaria control programmes and demonstrate that spatial repellents are useful for locations where people do not use nets for cultural reasons [34] or where vectors bite before people go to bed [35,36]. The mortality of mosquitoes induced by coils was as high as that of DDT. More than 60% of the mosquitoes collected from huts after exposure to coils died within 24 hours, having acquired lethal doses. This has implications for vector control programmes as it is thought that irritancy or excito-repellency of insecticides used on LLINs attenuates efficacy by preventing contact of mosquitoes with treated surfaces [37,38]. In this study it is shown that coils are capable of dispensing lethal doses of airborne insecticides and have the potential to reduce mosquito densities (m) and indirectly reduce chances that a mosquito would survive (p) long enough to become infectious. This study also shows that airborne pyrethroids reduce fitness of mosquitoes by reducing the number of eggs laid. Reduced fecundity is an indirect measure of pyrethroids on mosquito densities (m). However, further studies will be performed to investigate the combined impact (additional or deleterious) of indoor spatial repellents combined with LLINs on mosquito mortality and feeding success.

Among challenges facing malaria control, insecticide resistance could be considered top of the list. In this particular study area susceptibility of *An. arabiensis* mosquitoes is within the WHO set range of 80% - 97% at which resistance is suspected [39]. Therefore low mortality observed in the field could be attributed to slow emerging resistance [40-42]. A study carried out in Benin indicated that coils were effective against highly *kdr* resistant *Cx. quinquefasciatus quinquefasciatus* (Raphael Nguessan *pers. comm*). This indicates that spatial repellency may still provide protection where resistance has developed because airborne pyrethroids have an olfactory mode of action at low concentrations [43], different from the sodium channel target. These data warrant further investigation to see whether pyrethroid-resistant mosquitoes react differently to spatial repellents in ways that would affect vectorial capacity and malaria transmission.

The risk of mosquitoes being diverted to non-users of spatial repellents is likely to be increased if mosquitoes are prevented from feeding and continue host seeking [44]. A recent study has shown that topical repellents increase the proportion of mosquitoes to nearby non-users by approximately 4 times [45]. Nevertheless, the high toxicity of coils observed in the semi-field study might contribute to community protection. Toxicity coupled with the spatial activity of coils conferring protection in a defined area, may minimize the risk to non-users. In addition, almost half of the mosquitoes that left huts with mosquito coils and DDT died after 24 hours, consequently minimizing the population of mosquitoes that would be diverted to non-users within a community.

Nevertheless, it is necessary to improve delivery formats of airborne insecticides with the aim of expanding protection to a household or a community. In addition, it is essential to quantify the effect of using spatial repellents among non-users at different coverage levels and determine the implications on malaria transmission at a community level through large-scale trials before they are considered as a public health intervention.

Effectiveness of any vector control tool is influenced by whether or not it protects users against nuisance bites. Results from this study indicate that only Metofluthrin coils reduced house entry of *Cx. quinquefasciatus* mosquitoes by almost 28% in Lupiro village while DDT and Transfluthrin coils had no effect. The impact of all compounds on the entry of *Mansonia* spp. mosquitoes was outstanding (Table 4). All compounds reduced entry by more than 50%. It is necessary to develop spatial repellents that are equally effective against nuisance mosquito species in order to enhance compliance.

It should be noted that mosquito coils need to be used on a daily basis and produce smoke that could be harmful in long term exposure and might not be desirable to many people. The development of safer, effective, long lasting passive delivery formats is underway [46,47].

## Conclusions

It is critical to determine the impact of spatial repellents on malaria transmission. This study outlines several important entomological parameters that should be quantified in a proof of concept clinical trial in order to effectively determine the impact of spatial repellents on malaria epidemiology. In this study spatial repellents reduce human – vector contact and induce mortality, hence directly affect ma, m and p which are among the most important parameters of the vectorial capacity of a mosquito population. In addition, the role of spatial repellents in integrated approach of malaria control should be critically considered with an aim of complementing existing mainstream tools. Most available control tools, such as LLINs, require daily compliance by the user and may only be fully effective where malaria vectors still bite indoors late at night. Spatial repellents may be a suitable supplementary option where mosquitoes feed in the early evening and/or rest outdoors. In addition, because they render a given space mosquito free, they will protect multiple individuals in this space. The development of a passive spatial repellent that delivers the same mosquito control benefits of the mosquito coils tested in this study, but lasts for several weeks without the need for user compliance would contribute considerably to vector borne disease prevention.

## Competing interests

The authors declare that they have no competing interests.

## Authors’ contributions

SJM conceived the study; SBO, LML, MFM and SJM designed experiments. JM and AM designed and constructed the experimental huts and the semi-field system. SBO HN, RS, ES, MK, EM and MK conducted experiments. DR identified the mosquitoes to species level using PCR. SBO, LML, KK, MFM and SJM analysed the data. SBO drafted the manuscript in consultation with the other authors. All authors read and approved the final manuscript.
